# Corrections

**Published:** 1897-11

**Authors:** M. R. Leverson


### CORRECTIONS.

Owing to a miscarriage of the proofs the abstract of the testimony before the Royal British Commission in the October issue was published without being revised by Dr. Leverson.

The following literal errors should be corrected: Page 393, line 3, for "marked" read "masked." Page 395, Q. 994, for "By Dr. Saivey," read "By Dr. Savory." Page 398, line 8, for "380-52c" read "280-5, 2d." Page 402, line 17, for "Barne's" read "Baron's." Page 405, line 24, for "diseases" read "disease." Other revisions were intended, one of which was to add to the illustrations of Dr. Sweeting's self-contradictions, his answer to Q. 3,825, "We have had 148 people admitted" (admitted, *i. e.*, to the small-pox hospital) "with five or more marks," and Q. 3,832, "I should not regard him" (*i. e.*, a person having only one mark) "as being so fully protected as a person with five marks. He might, perhaps, be as fully protected against attack, but not against death by small-pox."

How were the 148 cases of small-pox of persons having five or more marks protected?

